# Benthic ecosystem functioning under climate change: modelling the bioturbation potential for benthic key species in the southern North Sea

**DOI:** 10.7717/peerj.14105

**Published:** 2022-10-26

**Authors:** Michael Weinert, Ingrid Kröncke, Julia Meyer, Moritz Mathis, Thomas Pohlmann, Henning Reiss

**Affiliations:** 1 Department for Marine Research, Senckenberg am Meer, Wilhelmshaven, Germany; 2 Faculty of Biosciences and Aquaculture, Nord University, Bodø, Norway; 3 Institute for Chemistry and Biology of the Marine Environment (ICBM), Carl von Ossietzky University, Oldenburg, Germany; 4 Institute of Coastal Systems, Helmholtz-Zentrum Hereon, Geesthacht, Germany; 5 Institute of Oceanography, University of Hamburg, Hamburg, Germany

**Keywords:** Benthos, Bioturbation index, Long-term variability, Species distribution models, Biomod2, Projections, Ecosystem management, Macrofauna

## Abstract

Climate change affects the marine environment on many levels with profound consequences for numerous biological, chemical, and physical processes. Benthic bioturbation is one of the most relevant and significant processes for benthic-pelagic coupling and biogeochemical fluxes in marine sediments, such as the uptake, transport, and remineralisation of organic carbon. However, only little is known about how climate change affects the distribution and intensity of benthic bioturbation of a shallow temperate shelf sea system such as the southern North Sea. In this study, we modelled and projected changes in bioturbation potential (BP_p_) under a continuous global warming scenario for seven southern North Sea key bioturbators: *Abra alba, Amphiura filiformis, Callianassa subterranea, Echinocardium cordatum, Goniada maculata, Nephtys hombergii*, and *Nucula nitidosa*. Spatial changes in species bioturbation intensity are simulated for the years 2050 and 2099 based on one species distribution model per species driven by bottom temperature and salinity changes using the IPCC SRES scenario A1B. Local mean bottom temperature was projected to increase between 0.15 and 5.4 °C, while mean bottom salinity was projected to moderately decrease by 1.7. Our results show that the considered benthic species are strongly influenced by the temperature increase. Although the total BP remained rather constant in the southern North Sea, the BP_p_ for four out of seven species was projected to increase, mainly due to a simultaneous northward range expansion, while the BP_p_ in the core area of the southern North Sea declined for the same species. Bioturbation of the most important species, *Amphiura filiformis* and *Echinocardium cordatum*, showed no substantial change in the spatial distribution, but over time. The BP_p_ of *E. cordatum* remained almost constant until 2099, while the BP_p_ of *A. filiformis* decreased by 41%. The northward expansion of some species and the decline of most species in the south led to a change of relative contribution to bioturbation in the southern North Sea. These results indicate that some of the selected key bioturbators in the southern North Sea might partly compensate the decrease in bioturbation by others. But especially in the depositional areas where bioturbation plays a specifically important role for ecosystem functioning, bioturbation potential declined until 2099, which might affect the biochemical cycling in sediments of some areas of the southern North Sea.

## Introduction

Benthic bioturbation is together with bioirrigation a key ecosystem process affecting biogeochemical cycles at the sediment-water interface and is, thus, of importance for benthic-pelagic coupling and functioning of marine ecosystem processes (Mermillod-Blondin, 2011; Kristensen et al., 2012; Griffiths et al., 2017; Zhang et al., 2019). This biotransport directly or indirectly reworks the sediments and consequently enhances the redistribution of organic matter, nutrients, and oxygen as well as the remineralisation of organic matter in the burrows (Jones, Lawton & Shachak, 1994; Lohrer, Thrush & Gibbs, 2004; Kristensen et al., 2012; Arlinghaus et al., 2021).

Sediments in continental shelf seas, where bioturbation seems to be most important (Solan et al., 2019), represent only 7% of the world’s oceans area, but are responsible for 52% of the global organic matter remineralization (Thrush & Dayton, 2002). The relative role of benthic biotransport compared to advection will vary with the environmental conditions (Snelgrove et al., 2018; Neumann et al., 2021), but up to half of the nutrients available to benthic primary producers of shelf seas are made available through benthic bioturbation and bioirrigation (Pilskaln, Churchill & Mayer, 1998; Wrede et al., 2017; Zhang et al., 2019; Neumann et al., 2021). Therefore, changes in distribution and composition of benthic bioturbators can significantly affect the marine ecosystem functioning and services (Snelgrove, 1999; Lohrer, Thrush & Gibbs, 2004).

In the marine realm, anthropogenic pressures, including climate change, fisheries and pollution, affect ecosystem integrity (Halpern et al., 2008), and human impacts have further increased over the last decade mainly due to climate forcing (Halpern et al., 2015). Thus, climate change is affecting environmental conditions and habitat suitability on large spatial scales with ramifications for species distributions, community structures and diversity patterns (Harley et al., 2006; Hoegh-Guldberg & Bruno, 2010; Poloczanska et al., 2013; Weinert et al., 2016), and consequently ecosystem processes and functioning (Gamfeldt et al., 2015; Nagelkerken & Connell, 2015; Griffiths et al., 2017). For example, Solan et al. (2004a) found that biodiversity loss generally coincides with bioturbation decline, but the magnitude and order of species loss depend on the species life traits and their resilience to the particular environmental impacts. In particular, sites with high macrofauna species richness and high community bioturbation potential (BP_c_) showed the highest decline in ecosystem functions, and the recovery of these sites with many sessile, slow-growing and long-lived species took much longer than in low diversity areas (Lohrer et al., 2010). Thus, climate-induced shifts of species and the resulting changes in the composition of benthic communities might eventually affect ecosystem processes, i.a. through altered bioturbation patterns. But not only climate affects the bioturbation, since Meyer et al. (2018) found a southern North Sea BP_c_ decline since the 1980s due to decreasing nutrient supply *via* rivers and decreasing primary production. This de-eutrophication process decreased abundance and biomass of species, but not diversity.

The North Sea, a continental shelf sea in the North-East Atlantic, is affected by multiple anthropogenic stressors with climate warming as the most important external driver (Halpern et al., 2008, 2015). The sea surface temperature in the North Sea increased by 1.5–2 °C since 1950 (Beare et al., 2002; Dulvy et al., 2008; Engelhard, Righton & Pinnegar, 2014), which is more rapidly than the global average (MacKenzie & Schiedek, 2007), and the northern European shelf seas can be considered as a “hot spot” of global warming (Holt et al., 2012). The bottom water temperature in the southern North Sea was projected to further increase by up to 5 °C until the end of the 21^st^ century (Mathis & Pohlmann, 2014; Mathis, Elizalde & Mikolajewicz, 2018; Mathis & Mikolajewicz, 2020). The temperature changes that occurred, resulted in distribution shifts of marine species in the North Sea documented for fish (Perry et al., 2005; Ehrich et al., 2007), plankton and benthic species (Beaugrand & Reid, 2003; Hiscock et al., 2004; Rees et al., 2007; Kröncke et al., 2011; Hiddink, Burrows & García Molinos, 2015). Most species respond to warming by a northward expansion of their distribution in the previously colder water. Future projections of 75 benthic species showed a latitudinal northward shift for 64% and suitable habitat loss for 65% of the species (Weinert et al., 2016). These species distribution shifts, especially when linked to changes in ecosystem processes, have the potential to also alter ecosystem functioning. Consequently, an assessment of climate change impacts on distribution and ecosystem processes at large spatial scales can offer important insights for ecosystem management and conservation planning (Reiss et al., 2015; Jones et al., 2016; Rilov et al., 2019; Weinert et al., 2021).

In our modeling approach, we combine species distribution modelling (SDM) and species bioturbation potential (BP). The latter is an index to estimate the species contribution to the biogenic mixing of sediments (Solan et al., 2004a) and combines the parameters biomass and abundance of species with the traits mobility and sediment reworking (Solan et al., 2004a, 2004b; Queirós et al., 2013). The BP of individual species populations (BP_p_) can be summed up to the community bioturbation potential (BP_c_). Previous studies showed, that BP_c_ represents a good proxy for important parameters in the sediment, such as oxygen concentration and consumption, ammonium and alkalinity fluxes, denitrification, total organic carbon and sediment mixing intensity (Solan et al., 2004a; Solan, Aspden & Paterson, 2012; Braeckman et al., 2014a; Morys, Powilleit & Forster, 2017; Gogina et al., 2018). The BP_c_ seems to be a good proxy for bioturbation distance, while it is limited in estimating the bioturbation attributes depth, activity and biodiffusive transport (Queirós et al., 2015). Nevertheless, the BP_c_ has been used as a proxy to quantify the consequences of climate-driven changes on ecosystem functioning in marine ecosystem (Solan et al., 2004a; Lohrer et al., 2010; Queirós et al., 2011).

Modelling the distribution of species and their projected shifts in response to climate change was only possible for a subset of species in the southern North Sea. Therefore, we selected key bioturbators, which are likely responsible for a large part of the total bioturbation in the southern North Sea (Wrede et al., 2017; Meyer, Nehmer & Kröncke, 2019). Wrede et al. (2017) found that 15 macrofauna species (out of 383 taxa) accounted for 75% of the bioturbation potential in the German Bight, and almost half of the BP_c_ was represented by only three species: the sea urchin *Echinocardium cordatum* (Pennant, 1777), the brittle star *Amphiura filiformis* (O.F. Müller, 1776), and the bivalve *Nucula nitidosa* Winckworth, 1930. In addition, we selected species based on their potential importance for bioturbation such as burrowing shrimp *Callianassa subterranea* (Montagu, 1808) and the bivalve *Abra alba* (W. Wood, 1802), or based on their wide distribution such as the polychaetes *Goniada maculata* Örsted, 1843, and *Nephtys hombergii* Savigny in Lamarck, 1818, see Dauwe, Herman & Heip (1998) and Gutow et al. (2020).

This study aims to assess the response of these seven benthic key bioturbators in the North Sea to projected bottom temperature and salinity changes for the years 2050 and 2099 (Mathis & Pohlmann, 2014) based on Intergovernmental Panel on Climate Change (IPCC) SRES scenario A1B. This study is a follow-up investigation using the same scenarios that were used for our previous studies on the spatial changes of macrofauna distribution in the North Sea (Weinert et al., 2016; Weinert et al., 2021). The bioturbation potential for each key species (BP_p_) and the total BP for all species selected in this study (BP_t_) were used as an indirect estimate for climate driven impacts on ecosystem functioning. The distribution models (biomod2) were first used to project species probability of occurrence (Thuiller et al., 2009), which provided the basis for a separate Random Forest (RF) regression model (Breiman, 2001), to model and project BP_p_ and BP_t_ for the North Sea. The main objective of this study was to project the distribution of the bioturbation potential under climate change as an indicator for expected changes in ecosystem functioning. We hypothesise that the key bioturbating benthic species differ in their projected response to climate warming and that this will be also translated into projected changes of their BP_p_.

## Materials and Methods

### Species data

The considered species data (presence, absence and species mean abundance per station, sampled with a Van Veen grab, 0.1 m^2^) were extracted from a data set collected for the EU-Project ‘Managing Fisheries to Conserve Groundfish and Benthic Invertebrate Species Diversity’ (MAFCONS) during the years 2003 and 2004. In total, 284 stations were sampled and used for the analyses. Not all countries which participated in the MAFCONS weighed the species individually. Therefore, the individual species mean wet biomass (g) were provided by Meyer et al. (2018) who compiled these data from different surveys in the study area. Our selection of the benthic key species *Abra alba*, *Amphiura filiformis*, *Callianassa subterranea*, *Echinocardium cordatum*, *Goniada maculata*, *Nephtys hombergii*, and *Nucula nitidosa* is based on previous studies with a focus on ‘ecosystem engineers’ and ‘bioturbation’ in the North Sea (Dauwe, Herman & Heip, 1998; Bouma et al., 2009; Braeckman et al., 2010; Birchenough et al., 2012; Braeckman et al., 2014b; Wrede et al., 2017; Meyer et al., 2018; Gutow et al., 2020). Details about the ecological and behavioural features and preferences of the species can be found in (Hartmann-Schröder, 1996; Ingle & Christiansen, 2004; Southward & Campbell, 2006; Zettler & Alf, 2021).

### Species bioturbation potential

The BP_p_ is an index to estimate the species contribution to the biogenic mixing of sediments and was first described by Solan et al. (2004a). It is calculated as follows:

$\rm BP_{p} = (B_{i}/A_{i})^{0.5}* A_{i} * M_{i} * R_{i}$

B_i_ and A_i_ are mean body size (biomass in grams) and mean abundance (individuals 0.1 m^2^) of species (i) at a sampled station, while M_i_ and R_i_ are life traits (mobility, reworking). The latter are categorical scores according to Solan et al. (2004a) and allocated for over 1,000 macrofaunal species in Queirós et al. (2013) describing the mobility from one (for organisms that live in fixed tubes) to four (free movement *via* burrow systems) and reworking from one (for epifauna) to five (for regenerators). The BP_p_ was log_10_ (x+1) transformed to down-scale large values. It was calculated separately for each species and sampled station. Finally, the total BP for all analysed key species (BP_t_) was calculated as follows:

$\rm BP_{t} = (BP_{p}1 + BP_{p}2 + ... + BP_{p}n)$

The BP_t_ in our study is not equivalent to the BP_c_ in other studies, because we only consider seven selected species and not the entire community although we assume that these species represent a large proportion of the community bioturbation potential. Furthermore, the BP_t_ was only calculated for southern North Sea, because species with a core distribution in the northern North Sea were not included. Thus, total community BP for the entire North Sea would be misleading when only based on southern species, while modelling the BP_p_ needs to include the possible expansion towards northern regions.

### Environmental parameters

The choice of environmental parameters is an important issue for the species distribution modelling. They can be grouped in three main categories: (1) limiting parameters that have eco-physiological influence, (2) natural and human-induced disturbances and habitat characteristics, and (3) resources, matter and energy consumed by species (Guisan & Zimmermann, 2000; Guisan & Thuiller, 2005). Six relevant environmental parameters were chosen (projected **mean bottom temperature** and **salinity** as well as **depth**, **mud content**, **median grain size** and **peak wave stress**), determining the spatial variability of the considered benthic ecosystem engineers (Callaway et al., 2002; Neumann, Ehrich & Kröncke, 2009; Reiss et al., 2010), to model and project BP_p_ and BP_t_ for the North Sea. The parameters were rasterized, with a resolution of 0.06 × 0.06 decimal degree and an extent of 60.4 north, 50.9 south, 3.1 west and 10.3 east (World Geodetic System 1984). Prior to the modelling process, raster cells covering land were excluded.

Mean bottom temperature (°C) and salinity of the simulated years 2001, 2050 and 2099 (Mathis & Pohlmann, 2014) were used to address climate change signals in the North Sea for the 21^st^ century. The annual means of volume-averaged temperature and salinity for 2001 are close to the climatological means for the period 1981–2000, so that the physical conditions in 2001 can also be regarded to represent mean conditions of the recent past. A global climate projection of the IPCC SRES scenario A1B was utilized to dynamically downscale potential climate change impacts for the North Sea to a meso-scale horizontal resolution of about 3 km (Mathis, Mayer & Pohlmann, 2013; Mathis & Pohlmann, 2014). Projections of mean bottom temperature for February and June were applied to consider seasonality and because the second quarter of the year was ecologically most affected by previous winter temperatures (Kröncke et al., 1998; Kröncke, Reiss & Dippner, 2013). After cold winters, a higher percentage of arctic-boreal species were found, whereas a positive North Atlantic Oscillation (NAO) index linked to warmer conditions facilitate an increase of species distributed in the southern North Sea (Kröncke, Reiss & Dippner, 2013). Simulations for the years 2001, 2050 and 2099 were chosen to project both, changes until mid-century and the end of the century to show changes over time. In general, mean bottom temperature for February and June was projected to change between −1.9 and 5.4 °C. An increase of winter temperatures (February) by 3 °C (2050) and by 3.4 °C (2099) were projected for the Danish coast, Skagerrak, central North Sea and German Bight (Supplemental Material S1). A decrease of winter temperatures by 0.5 °C (2050) was projected for the Southern Bight. During summer (June), a temperature increase by 1.8 °C (2050) and by 5.4 °C (2099) was projected for the southern North Sea (to a depth of 30–40 m) and to some degree for the Dogger Bank. A decrease of summer temperatures by 1.9 °C was projected for the Skagerrak and partly in the northern North Sea. Mean bottom salinity was projected to increase in summer by 1.0 (2050) and 1.4 (2099) for the Dutch and Belgian coast, the German Bight (only 2050) and the northern North Sea. In contrast, salinity was projected to decrease by 1.7 (2050) and 1.5 (2099) for the German Bight, the Dutch coast, the Oyster Ground and Skagerrak (Fig. S1a-i; Mathis & Pohlmann, 2014).

Depth data, with a resolution of one arc-minute (1.852 km), were derived from the General Bathymetric Charts of the Oceans (GEBCO) global bathymetry data set from the British Oceanographic Data Centre (GEBCO, 2003). Mud content and median grain size were collected during the North Sea Benthos Project (NSBP 2000) and the project Managing Fisheries to Conserve Groundfish and Benthic Invertebrate Species Diversity (MAFCONS). Data of peak wave stress, with a resolution of about 12 km, were provided by the Proudman Oceanographic Laboratory (Liverpool, UK), generated with the help of a 3-dimensional hydrodynamic model (Davies & Aldridge, 1993). Peak wave stress was calculated from a 1-year model run, covering the period September 1999 to September 2000.

More details regarding the environmental parameters can be found in (Reiss et al., 2011; Mathis & Pohlmann, 2014; Weinert et al., 2016). In contrast to bottom temperature and salinity, all other environmental parameters were kept constant over time.

### The modelling approach

The spatial distribution of the BP_p_ for the seven species in the North Sea was modelled for the year 2001. The impact of climate change was projected for the years 2050 and 2099. For the analyses, the R environment version 3.5.1 (R Core Team, 2018) was used and applied in two steps: First, the R package ‘biomod2’ version 3.3-7 (Thuiller et al., 2009; Thuiller et al., 2015) was used to generate ensemble and consensus species distribution models, to model and project the probability of occurrence as well as presence and absence for each species. Biomod2 has the advantage, to combine several model algorithms to reduce uncertainties derived from the usage of different algorithms (Thuiller et al., 2009) and to use a consensus model based on weighted evaluation indices (Marmion et al., 2009) to separate between poor and reliable model results. Second, RF algorithm (Breiman, 2001) was chosen and the R packages ‘randomForest’ version 4.6-14 (Liaw & Wiener, 2002) and ‘caret’ version 6.0-80 (Kuhn et al., 2018) were applied to model and project the BP_p_. For the second step, we followed the methodological approach of Hill et al. (2017).

### Modelling and projecting the BP_p_

For the first step, the R package ‘biomod2’ version 3.3-7 (Thuiller et al., 2009; Thuiller et al., 2015) was used to apply four model algorithms, which represent different approaches commonly used (*i.e*. regression models, classification trees and machine learning models): Generalized Linear Model (GLM) (McCullagh & Nelder, 1989), is an extension of the classical linear regression model, that allows the application of different distributed data like Gaussian, Poisson, Binomial, or Gamma. “GLMs are based on an assumed relationship (link function) between the mean of the response variable and the linear combination of the explanatory variables” (Guisan, Edwards & Hastie, 2002). Multiple Adaptive Regression Splines (MARS) (Friedman, 1991), is a flexible nonparametric regression model that combines piecewise linear functions (splines) and recursive partitioning to handle both linear and nonlinear relationship between dependent and independent variables. A forward/backward stepwise approach is used to generate the optimal model *via* cross-validation. Generalized Boosting Model (GBM) (Ridgeway, 1999), is an additive regression model consisting of many weak regression or classification trees, fitted in a forward, stagewise fashion (boosting) to estimate the final robust model (Elith, Leathwick & Hastie, 2008). Random Forest (RF) (Breiman, 2001; Prasad, Iverson & Liaw, 2006) is a classification and regression model, where classification trees (500–2,000) are drawn to maximum size without pruning based on resampled subsamples with replacement (bootstrapping) using a randomised subset of predictors. The average of all trees is calculated to aggregate the results. In this study, the default model options for the RF classification model were changed. The parameter ‘mtry’ (*i.e.*, the number of variables randomly sampled at each node) was tested with values of 2, 3, 4 and 5 following Darr, Gogina & Zettler (2014) and the parameter ‘nodesize’ (*i.e.*, the minimum size of nodes) was changed to 1, the default value for classification models (Liaw & Wiener, 2018). Thus, for each species, four model options were tested and the model with the highest evaluation index (mean AUC and TSS) was used to generate the RF species distribution model.

For model calibration and evaluation, the species occurrence data were randomly split into two subsets, 70% used for training and 30% for testing the model. Independent data were missing, thus 20 replicate runs were performed per algorithm (a sort of cross-validation) to account for algorithm variability (Thuiller et al., 2015). The area under the receiver operating characteristic (ROC) curve, often described as Area Under the Curve (AUC), and the True Skill Statistic (TSS) were used to evaluate the model results (Fielding & Bell, 1997; Allouche, Tsoar & Kadmon, 2006). Both measures are based upon the error matrix (true and false positive and negative rate). The AUC varies between 0 and 1, it is a threshold and prevalence independent index representing the relationship between the true positive rate (sensitivity) and the corresponding proportion of the false positive rate (1 – specificity). The TSS ranges between −1 and 1, it is a prevalence independent index and is defined as the sum of true positive rate (sensitivity) and true negative rate (specificity) minus 1, thus omission and commission errors (false negative rate, false positive rate) were accounted for (Allouche, Tsoar & Kadmon, 2006). Thresholds were applied for both evaluation indices (AUC ≥ 0.7 and TSS ≥ 0.4) to separate between poor and reliable replicate runs (Araújo et al., 2011). For each species only robust replicate runs were included in further analyses. The robust replicate runs were merged for each model algorithm in an ensemble model, separately for each species and year (2001, 2050 and 2099). An ensemble model is basically the mean of all projections with a single model algorithm. Thus, for each species analysed, three ensemble models (2001, 2050 and 2099) were generated with each model algorithm. Finally, robust replicate runs of all model algorithms were merged in a consensus model, again separately for each species and year. A consensus model is the weighted mean of the projections carried out with all model algorithms. The evaluation indices were weighted, so that a model result with a high evaluation index was more important in the consensus model. Finally, cut-off levels were applied to convert the consensus distribution maps of species probability of occurrence into presence/absence to adapt and apply the zero-inflated model approach from Savage, Lawrence & Squires (2015). For an explanation see the second step in the analyses. The applied cut-off levels maximised AUC and TSS, thus the most reliable distribution maps were generated (Jiménez-Valverde & Lobo, 2007; Barbet-Massin et al., 2012).

For the second step, RF algorithm was used and the R packages ‘randomForest’ and ‘caret’ were applied, to generate a separate RF regression model for each species. Thus, for each sampled station the calculated BP_p_, the modelled and projected probability of species occurrence from the first step of the analyses and the values from the environmental parameters were used to model and project the distribution of the BP_p_ of the seven benthic key species in the North Sea under climate change. The evaluation metrics root-mean-square error (RMSE) and mean absolute error (MAE) were calculated by 10-fold cross-validation and indicate the average error of a model (Chai & Draxler, 2014). Both parameters have the same approach and measure the differences between prediction and actual observation. They indicate the average error of a model given in the same unit as the response variable, in this study the BP_p_ per raster cell (0.06 × 0.06 decimal degree). While the RMSE gives more weight to large residuals and is thus more sensitive to outliers in a small sample size (*n* < 100), the MAE gives equal weight to small and large residuals (Chai & Draxler, 2014). Both evaluation metrics can range from zero to infinite, where zero means no error of the model. The lower limit of 50 data points (sampled species presence) was defined to apply 10-fold cross-validation for the evaluation, which gives an average of five nonzero data points per fold for six out of seven species. For the burrowing shrimp *C. subterranea* less than 50 data points were available. Thus, 6-fold cross-validation was chosen to maintain the average of five nonzero data points per fold. A detailed description of the method is given in Hill et al. (2017).

### Analyses of the bioturbation potential under climate change

The zero-inflated model approach from Savage, Lawrence & Squires (2015) was adapted and applied to generate the final BP_p_ distribution maps for the years 2001, 2050 and 2099. Therefore, each BP_p_ distribution map was masked with the correspondent binary distribution map of species presence/absence. Thus, BP_p_ values were assigned only for raster cells, which were modelled and projected as presence in the first step of the analysis. Vice versa, zero was assigned for raster cells, which were modelled and projected as absence. Afterwards, the BP_p_ values in the remaining raster cells were summed up to generate the BP_p_ in the North Sea for each year individually. This approach prevents over-projection and enables to generate reliable BP_p_ distribution maps. Finally, the BP_p_ distribution maps were added separately for the years 2001, 2050 and 2099 to calculate the cumulative total sum of the BP and generate the BP_t_ distribution maps. Bioturbation potential gain or loss was calculated by comparing the total BP_p_ and BP_t_ between the years 2001, 2050 and 2099. A general work-flow of the two-step approach can be found in Supplemental Material S2.

## Results

### Model statistics

The selected species distribution models above the applied thresholds showed a good model performance with AUC values between 0.72 and 0.8 and TSS values between 0.43 and 0.56 (Table 1). They were used to generate the final consensus models of species probability of occurrence as well as the binary distribution maps of species presence and absence. The consensus models are based on all applied model algorithms (GLM, GBM, MARS, RF) for five out of seven species. For the other two, it is a consensus model based on GLM, GBM and MARS (*N. hombergii*), as well as an ensemble model based on GLM only (*G. maculata*; Table 1). For the second step of the analyses, the model performance of the BP_p_ distribution modelling with RF was evaluated with the parameters MAE and RMSE. Both parameters showed low values (MAE 0.06-0.15, RMSE 0.15-0.39) and the MAE was about two to three times smaller than the RMSE for the models of all species (Table 1), both indicating relatively small differences between prediction and actual observation. In addition, the MAE and RMSE were calculated in per cent for each species to put them in relation to the calculated mean BP_p_ per raster cell, based on the sampled data. The prediction of BP_p_ per raster cell was good. An error of <3% (MAE) was predicted for the models of all species and <5% and <8% (RMSE) for the models of six and one species, respectively (Table 1).

**Table 1:** Overview of the observed species presence and absence, the included model algorithms, the model evaluation parameters as well as the importance of environmental parameters in the model. Overview of the observed species presence and absence (p/a), the amount of models used to build the final consensus model with included models across all model algorithms, the included algorithms (with ^1)^GLM, GBM MARS and RF, ^2)^GLM, ^3)^GLM, GBM and MARS) as well as the mean (m) and standard deviation (SD) of the evaluation parameters Area Under the Curve (AUC) and True Skill Statistic (TSS). The Random Forest regression model was evaluated with the indices mean absolute error (MAE) and root-mean-square error (RMSE). Both indices were also calculated in percent to present the average error of mean BP_p_ per raster cell. Results of the relative importance of environmental parameters (%) in the model (black = temperature June, orange = temperature February, sky blue = salinity June, yellow = depth, blue = median grain size, vermillion = mud content, reddish purple = peak wave stress).

Species	p/a	Models	AUC		TSS		MAE (%)	RMSE (%)	Variable importance (%)
			m	SD	m	SD			
*A. alba*	53/231	55^1)^	0.77	0.01	0.5	0.02	0.09 (2.25)	0.25 (7.38)	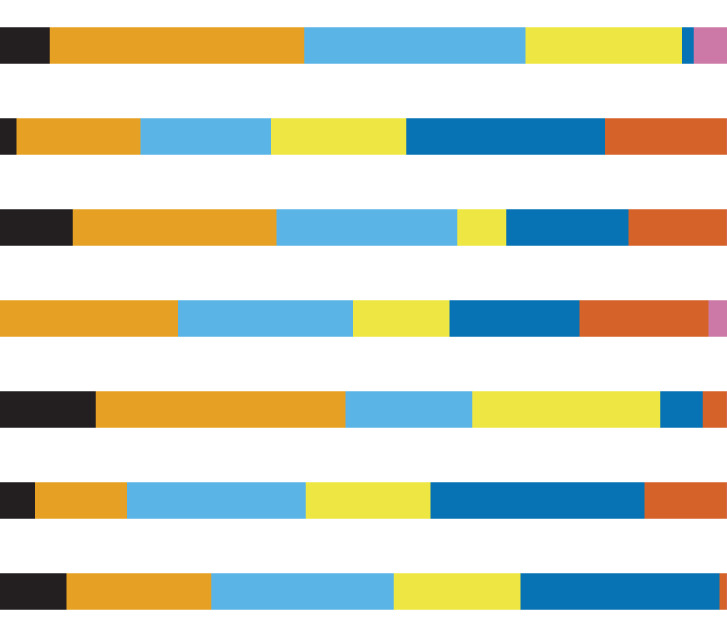
*A. filiformis*	132/152	14^1)^	0.74	0.02	0.43	0.03	0.15 (0.28)	0.38 (0.94)	
*C. subterranea*	35/249	68^1)^	0.8	0.005	0.55	0.04	0.10 (0.49)	0.23 (1.30)	
*E. cordatum*	116/168	24^1)^	0.72	0.004	0.44	0.02	0.14 (0.08)	0.39 (0.28)	
*G. maculata*	145/139	5^2)^	0.73	0.01	0.43	0.03	0.06 (1.55)	0.16 (4.72)	
*N. hombergii*	99/185	14^3)^	0.72	0.001	0.43	0.02	0.06 (0.77)	0.15 (2.21)	
*N. nitidosa*	53/231	77^1)^	0.8	0.009	0.56	0.02	0.11 (1.20)	0.31 (4.07)	

### BP_p_ and BP_t_ potential gain and loss

**Figure 1:**
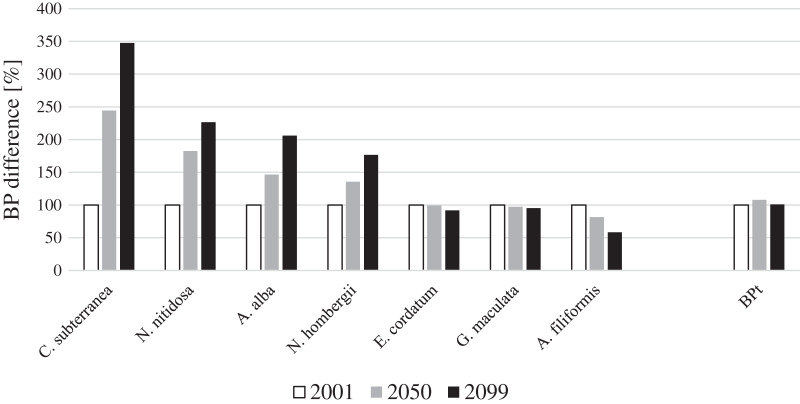
Species and total BP gain and loss in percent for the years 2001, 2050 and 2099 for the North Sea (note: BP_t_ was calculated for the southern North Sea only). The calculated BP_p_ and BP_t_ for the year 2001 were set to 100%. The gain and loss of BP_p_ and BP_t_ in 2050 and 2099 were calculated in relation to BP_p_ and BP_t_ in the year 2001.

**Figure 2:**
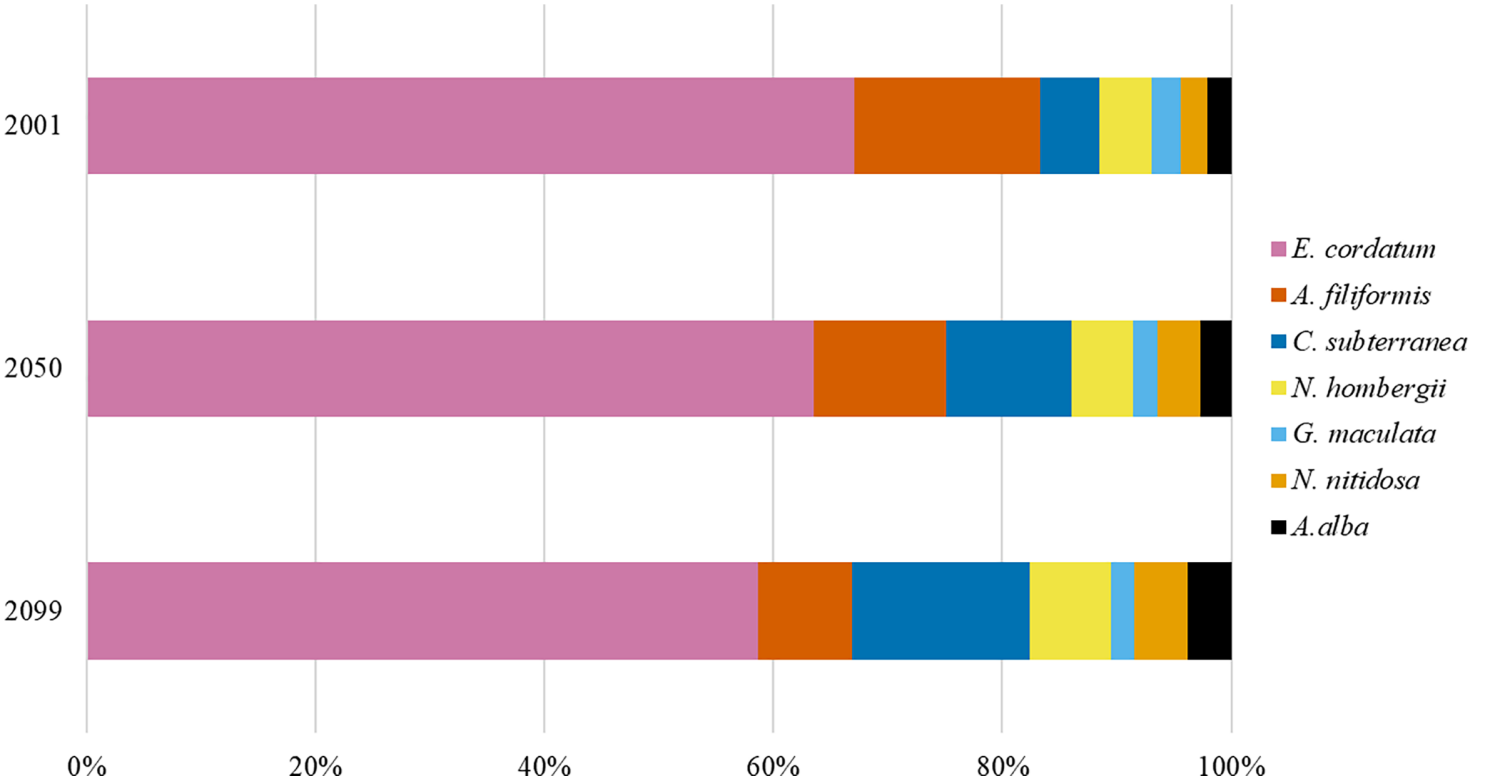
The relative contribution of BP_p_ to BP_t_ of the southern North Sea for the years 2001, 2050 and 2099.

For four key species BP gain and for three key species BP loss was projected until 2099 in the entire North Sea (Fig. 1). Continuous BP_p_ gain until the end of the 21^st^ century was projected for the bivalves *N. nitidosa* (113%) and *A. alba* (198%), the bristle worm *N. hombergii* (176%) and the burrowing shrimp *C. subterranea* (246%) (Fig. 1). The predicted BP_p_ gain from 2050 to 2099 was even higher for *A. alba* and *N. hombergii*, as for the period from 2001 to 2050. However, together the species contributed only 12% (2001), 20% (2050) and 28% (2099) to BP_t_ (Fig. 2). In contrast, BP_p_ loss was projected for the brittle star *A. filiformis* (−42%), the bristle worm *G. maculata* (−7%) as well as the sea urchin *E. cordatum* (−8%) from 2001 to 2099 (Fig. 1).

Overall, BP_t_ for the southern North Sea only was projected to increase slightly until 2050 (+8%) and decreased slightly again until 2099 (Fig. 1). The sea urchin *E. cordatum* was the most important bioturbator and was projected to be responsible for over 60% of BP_t_ in all the analysed years (Fig. 2). The relative contribution of the sea urchin *E. cordatum* decreased moderately over time and accounted for 71% (2001), 66% (2050) and 62% (2099) of BP_t_ (Fig. 2). In contrast, the burrowing shrimp *C. subterranea* was predicted to account for the highest proportional gain (it contributed to 5%, 11% and 15% of BP_t_ modelled for 2001, 2050 and 2099, respectively) and the brittle star *A. filiformis* for the highest proportional loss (it contributed to 15%, 12% and 8% of BP_t_) (Fig. 2). The bristle worm *N. hombergii* was predicted to almost double the proportional amount of BP_t_ from 2001 (4%) until 2099 (7%) (Fig. 2).

### Spatial distribution of the bioturbation potential

**Figure 3:**
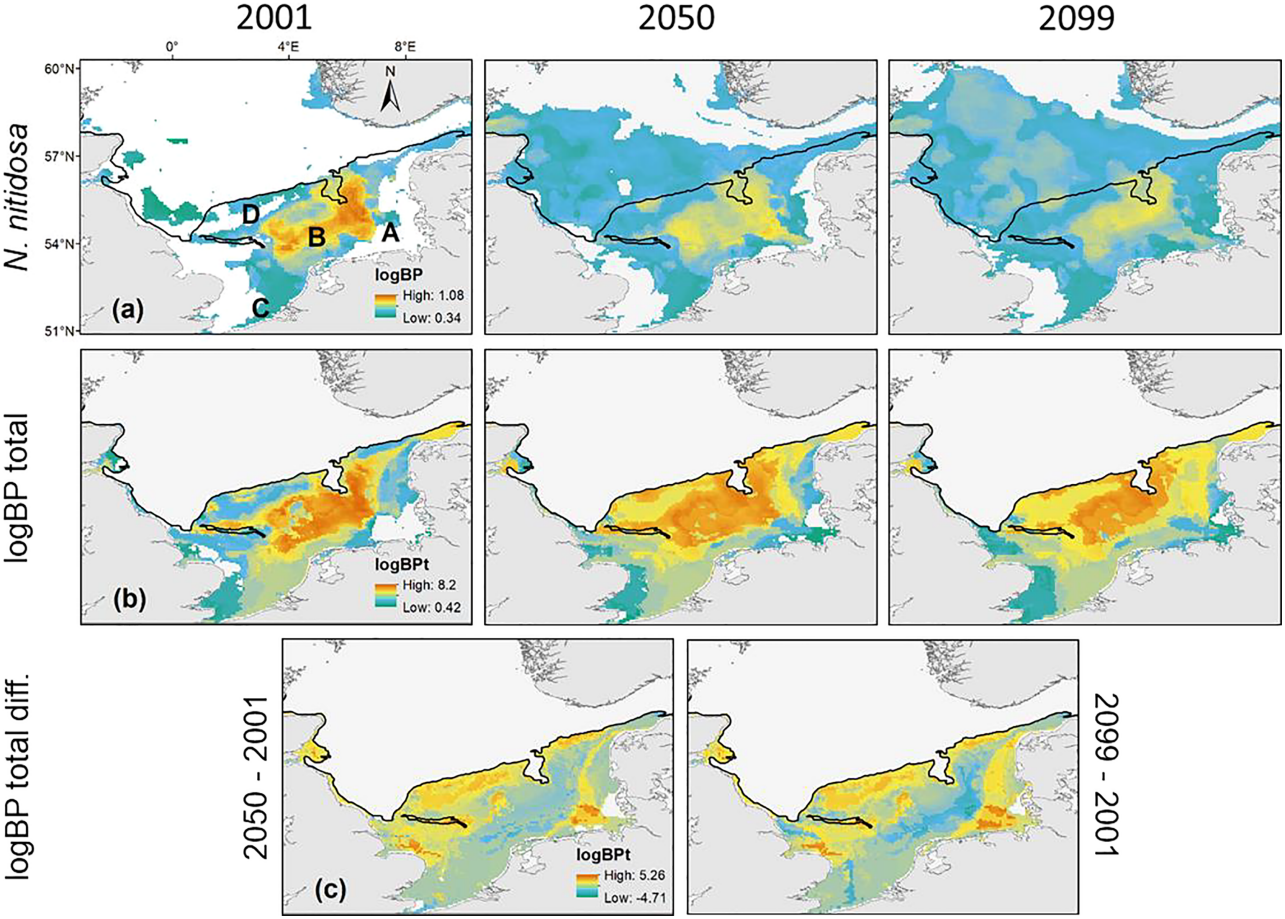
The projected and predicted spatial distribution of the analysed BP_p_ for the species *Nucula nitidosa* (A), BP_t_ (B) and BP_t_ difference (C) between 2001 and 2050 as well as 2001 and 2099 in the North Sea. Maximum and minimum of the BP_p_ and BP_t_ were individually adapted in the figures (all values given as log_10_). Bold letters (A) show the regions, we refer to in the text: A = German Bight, B = Oyster Ground, C = Southern Bight, D = Dogger Bank. Black line: 50 m depth contour.

Maximum values of BP_t_ per raster cell were projected for the southern North Sea only (Fig. 3), because species with a core distribution in the northern North Sea were not included. The highest BP_t_ was projected for the Oyster Ground and the southern edge of the Dogger Bank. Around this main distribution area, rather low values of BP_t_ were projected for the inshore regions of the southern and eastern North Sea, and the southern Bight (Fig. 3). The predictions showed, that BP_t_ increases moderately until 2050 (+8%) and decreased again until 2099 (−7%, Fig. 1). Nevertheless, the main distribution areas of the species BP_p_ were predicted to shift more and more to the north (see below), which goes along with a decrease of BP_t_ per raster cell in the southern North Sea (Fig. 3). This decrease was specifically pronounced in the regions with the highest BP_t_, *e.g.*, the Oyster Ground, although overall distribution of BP_t_ values in the southern North Sea remain relatively constant over time (Fig. 3).

**Figure 4:**
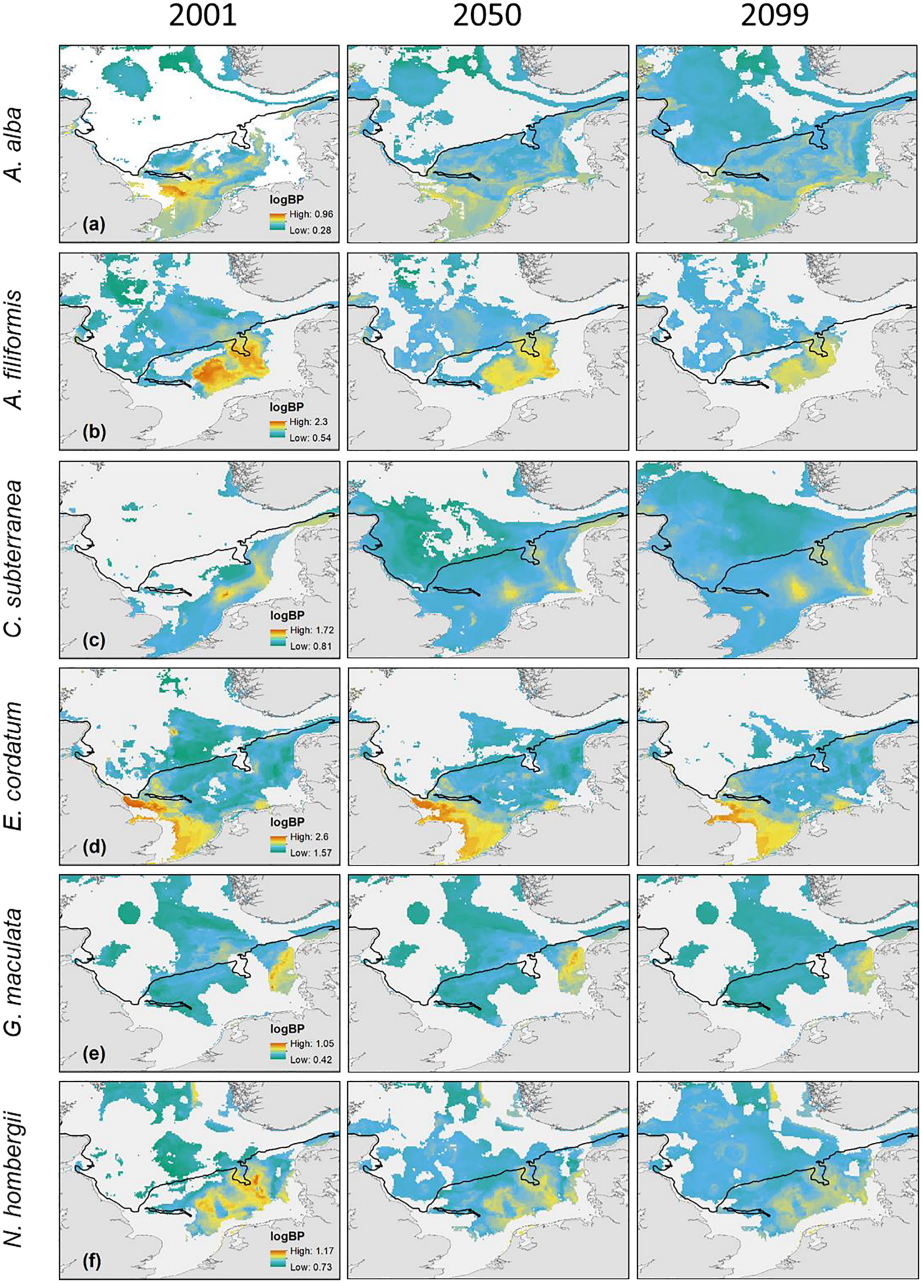
The projected and predicted spatial distribution of the analysed BP_p_ for the species *Abra alba* (A), *Amphiura filiformis* (B), *Callianassa subterranea* (C), *Echinocardium cordatum* (D), *Goniada maculata* (E) and *Nephtys hombergii* (F) in the North Sea. Maximum and minimum of the BP_p_ were individually adapted in the figures (all values given as log_10_). Black line: 50 m depth contour.

The main distribution area with high values of BP_p_ of the different species in the southern North Sea was projected for the Oyster Ground (*A. filiformis*, *C. subterranea*, *N. hombergii*, and *N. nitidosa*), the southern Bight (*A. alba, E. cordatum*) and German Bight (*G. maculata*) (Figs. 3 and 4). Most species did not only show increase or decrease of BP values, but an expansion towards the northern and eastern North Sea (Figs. 3 and 4). Predictions of gain showed an increase of BP_p_ specifically in the northern North Sea for most species, but again related to a decrease of the maximum values of BP_p_ in the southern North Sea (Figs. 3 and 4). Predictions of BP_p_ loss go along with a decrease in the main distribution areas, especially for the brittle star *A. filiformis* (Fig. 4).

## Discussion

Global warming is regarded as one of the most important anthropogenic drivers of ecosystem change, causing shifts in the latitudinal and longitudinal distribution of species due to changing environmental conditions and habitat suitability (Parmesan & Yohe, 2003; Thuiller, 2004; Lenoir et al., 2008; Chen et al., 2011; Hiddink, Burrows & García Molinos, 2015; Göransson, 2017). Since the beginning of the 20^th^ century, rapid and profound changes in species composition and diversity of marine ecosystems were found (Doney et al., 2012), with potential ramifications for ecosystem processes and functioning (Poloczanska et al., 2013). Predicting such responses to future climate change are thus essential for an effective management and conservation of biodiversity (Hannah, Midgley & Millar, 2002; Weinert et al., 2021).

Recent studies highlight the importance of large scale (Gogina et al., 2017; Meyer, Nehmer & Kröncke, 2019) and cross-territorial studies on spatial patterns of bioturbation (Gogina et al., 2020), which makes the combination of spatial distribution models and climate change scenarios conducted in this study particularly important for predicting changes in bioturbation patterns.

In this study we investigated the projected changes of bioturbation as an important ecosystem process in soft-bottom benthic communities in response to climate driven changes in sea bottom temperature and salinity. By using the bioturbation potential (BP) as a proxy, we found minor changes in the overall BP_t_ in the southern North Sea based on seven key species until the end of the 21^st^ century. Nevertheless, the relative contribution of the different species to the BP_t_ changed with a northward expansion of some species and a reduction of bioturbation in the core southern distribution areas, for example due to response of the main bioturbator brittle star *Amphiura filiformis*.

### A temperature induced northward shift of southern North Sea bioturbators

Among the consequences of anthropogenic climate change, the rapid increase of sea surface temperature (SST) is likely the main factor influencing marine ecosystems to date (Hiddink, Burrows & García Molinos, 2015; Kröncke et al., 2019; Moore & Smale, 2020; Pilotto et al., 2020). Shallow coastal ecosystems such as the southern North Sea are drastically influenced by an increase in mean SST of 1.5–1.8 °C since 1950 (Beare et al., 2002). For the North Sea Mathis & Pohlmann (2014) projected an approximately linear increase in SST of about 2 °C per 100 years for the emission scenario A1B, with an increase in the near coastal zone of up to 5 °C (Schrum et al., 2016). A similar increase was found for the bottom temperatures used in this study for the southern North Sea with predominantly mixed water masses, while changes in bottom temperature in the stratified northern North Sea were somewhat less pronounced (Mathis & Pohlmann, 2014; Weinert et al., 2016). The changes in projected bottom salinity (Mathis & Pohlmann, 2014) were only moderate until 2099 (−1.7 to 1.4; Fig. S1l, S1o) and we assume that salinity changes have a minor effect on the distribution of the species and thus changes in species BP. Therefore, we focus our interpretation in the following on temperature changes as one of the most important drivers of distribution changes in the North Sea, although interactive effects with salinity are also conceivable.

In the present study, a latitudinal northward shift in BP_p_ was found for four out of seven species. This is in line with the results of Weinert et al. (2016), who found a latitudinal northward shift of 64% of the benthic species of the North Sea. All four species, namely *C. subterranea*, *A. alba, N. nitidosa* and *N. hombergii*, have in common that the BP_p_ in the core area of the southern North Sea decrease, while the overall spatial distribution expands and BP_p_ increases in the more peripheral areas of the North Sea.

In terms of direction, for abundances of the mud shrimp *C. subterranea* a southward shift and a habitat loss of 31% was predicted by Weinert et al. (2016). In the present study we found a decrease in BP_p_ in the core region (Oyster Ground, German Bight) from 2001 to 2099, but in combination with wider distribution of low BP_p_ intensities. This spatial expansion though leads to a remarkable overall increase in BP_p_ of *C. subterranea* of over 300% for the entire North Sea from 2001 to 2099. *C. subterranea* can be found with the highest biomass in areas with a mud content of >50% (Gutow et al., 2020), while higher abundances were found on a wide range of sediments (Coleman & Poore, 1980; Gutow et al., 2020). Thus, our results indicate that negative effects on the distribution and the concerning BP_p_ due to increasing temperatures may be compensated by the broad tolerance to environmental conditions of some species. However, this tolerance remains uncertain for *C. subterranea* with the strong preference of large adults for muddy sediments, which are not ideally represented in the environmental settings of our model.

The widespread predatory polychaete *N. hombergii* inhabits predominantly sediments containing fine sands with a mud content up to 20% in shallow waters (>30 m) and has a core distribution in the German Bight (Meißner, Darr & Rachor, 2008). Beukema (1990) stated that increasing winter temperatures may increase the abundance of *N. hombergii* in coastal and Wadden Sea areas, because it is negatively affected by cold winters. Indeed, we also found an increase of BP_p_ for the entire North Sea with warming, but a rather opposing effect on the bioturbation of *N. hombergii* in the southern North Sea, where BP_p_ decreased in the core distribution area. The temperature and salinity induced northward shift seemed to be amplified by the broad habitat requirements concerning sediment structure and adaptable feeding habits, while the decrease in the south indicates a warming beyond the species tolerance levels.

For the small bivalve species *A. alba* and *N. nitidosa* Weinert et al. (2016) predicted a habitat loss over 50% with distributional shift in north-western direction for *N. nitidosa* and in north-eastern direction for *A. alba* due to the expected temperature and salinity changes. Similarly, in the present study locally high BP_p_ of both species decreases, while the overall BP_p_ increased through a northward range expansion. They are detritus feeder and inhabit muddy and fine-grained sediments (Kröncke & Bergfeld, 2003; Van Hoey, Degraer & Vincx, 2004; Van Hoey, Vincx & Degraer, 2007; Kröncke et al., 2011). While *N. nitidosa* is not sensitive to cold winters, *A. alba* showed a decline or complete absence after cold winters such as in 1995/96. However, both species were found in higher densities after mild winters (Kröncke & Bergfeld, 2003; Reiss, Meybohm & Kröncke, 2006; Van Hoey, Vincx & Degraer, 2007), explaining the temperature-driven changes in our future projection.

An almost stable distribution with minor changes in BP_p_ were found for the polychaete *Goniada maculata* and the echinoderm *Echinocardium cordatum*. The latter species is actively reworking fine sediments and was found to be one of the most important bioturbators in the North Sea (Kröncke et al., 2004; Wrede et al., 2017). The relatively stable BP_p_ of *E. cordatum* with only a slight decrease is partly in agreement with results of Weinert et al. (2016), who found hardly any shift in the core distribution over time, but a loss in suitable habitat until 2099 for the species. The latter seem to be contradictory to our results, since the overall BP_p_ has not changed over time, but the BP_p_ values distributed more evenly over the shrunken distribution area of the species in 2099 (Fig. 4).

In contrast, the brittle star *Amphiura filiformis* revealed a loss in BP_p_ by 41% in particular in the south eastern North Sea. It burrows in sediments with moderate mud content and is as interface feeder in often stratified areas with low organic matter supply (Kröncke et al., 2004). Our result seems surprising since *A. filiformis* is an eurytherm species also distributed in southern regions, which indicates tolerance of warmer conditions. However, its core distribution is in the temperate and boreal regions (Kaschner et al., 2019) and similar to the other species with decreasing distribution in the southern North Sea, the projected warming of up to 5 °C in the southern North Sea seem to exceed the temperature tolerance of the species.

### Changes in total bioturbation potential (BP_t_) and ecosystem functioning

Changes in water temperature (here SST) and de-eutrophication were found to be main drivers for past long-term changes in the spatial community bioturbation potential (BP_c_) variability in the southern North Sea, (Meyer & Kröncke, 2019; Meyer, Nehmer & Kröncke, 2019). While the BP_c_ is representing the community bioturbation potential (Solan et al., 2004a), we used and defined the total BP (BP_t_) as a sum for the selected species to represent the summative response of the main bioturbators to warming. This can be justified by the finding that relatively few species account for most of the bioturbation potential for example in the German Bight (Wrede et al., 2017) and that the loss of single key bioturbators can have substantial consequences for ecosystem functioning (Lohrer, Thrush & Gibbs, 2004). Due to this focus on the selected species, diversity effects are not represented in the total BP. For example, it was shown that the importance of bioturbation and bioirrigation for biogeochemical cycling is reduced by interspecific competition for food or space (Hale et al., 2014). Thus, weak bioturbators, that were not considered in this study, could have a modifying effect on bioturbation and the resulting ecosystem functioning due to species interactions in a diverse community. However, these interactive effects wouldn’t been fully represented by the BP_c_ as a static trait-based indicator either.

Considering the analysed southern North Sea key bioturbators, the BP_t_ remained relatively stable until 2099 mainly because of opposing trends in BP_p_ among the species described above (Fig. 1). A high functional redundancy in the southern North Sea was already found for the BP (Meyer & Kröncke, 2019) and other biological traits (Shojaei et al., 2021). This compensational effect based on changes in the distribution of species might buffer against climate change impacts on ecosystem functions, but it remains unclear if and to what extent this is actually the case, in particular under the combined influences of other anthropogenic stressors (*e.g.*, pollution, sand mining and dredging, bottom-contacting fishery). Some important bioturbators with a high contribution to the BP_c_ (based on their traits, biomass and abundance) such as *A. filiformis* or *N. nitidosa* were found to contribute comparatively little to the expressed biogeochemical cycling and sediment turnover (Wrede et al., 2017). Thus, realised and potential bioturbation might differ substantially and species might not be replaceable due to nuanced differences in activity (Hale et al., 2014; Queirós et al., 2015). Other species within the same functional guild but inhabiting southern warmer waters might actually compensate a loss in potential bioturbation when expanding their distribution range into the North Sea (Neumann et al., 2013; Zettler et al., 2018). While this aspect was not addressed in the present study, distribution modelling in general could indeed assist in investigating these large scale impacts of distributional changes and the role of non-native species (Reiss et al., 2015).

However, we found changes in the spatial distribution patterns and regional quantity of the BP_p_ on species level in the southern North Sea (Fig. 2), which is also reflected in the spatial changes of the BP_t_. Several species showed a decrease in BP_p_ over time in the core distribution areas in the south (Figs. 3 and 4), while a moderate increase was observed in the peripheral and northern areas. The predicted long-term spatial changes of the present study are in line with the long-term trends in BP_c_ of macrofauna communities in the southern North Sea since the 1980s, which were related to SST increase mediated by the de-eutrophication processes (Meyer & Kröncke, 2019; Meyer, Nehmer & Kröncke, 2019) and possibly the decrease in primary production, *e.g.*, (Capuzzo et al., 2018; van Beusekom et al., 2019; Desmit et al., 2020). In our study, a decline in BP_t_ was mainly projected for the depositional areas of the southern North Sea with predominantly fine, muddy sediments. In these less permeable sediments, bioturbation and bioirrigation by macrofauna are specifically important for the biogeochemical cycling in sediments. In contrast, in the more permeable sandy sediments, where we projected an increase of BP_t_ (*e.g*. the Dogger Bank), physical processes such as pore-water advection are much more important than biotransport (Neumann et al., 2021). Thus, BP might decline especially in areas where it is most important for biogeochemical cycling and ecosystem functioning.

### Caution in the use of BP

While we found indications of bioturbation changes until the end of the 21^st^ century, there are some limitations in the use of theoretical BP_p_ as a predictor for bioturbation activities and as a predictor for expected changes in ecosystem functioning. The theoretical BP_p_ (Solan et al., 2004b; Queirós et al., 2013) is currently the only proxy for using existing data as a basis for a large-scale statistical modelling of bioturbation, because North Sea wide field measurements are not available. The BP_c_ successfully predicts the distance of particle transport (Queirós et al., 2015), while the prediction of bioturbation activity, depth, and the biodiffusion coefficient is limited. However, comparisons of the theoretical BP_c_ with the results from experimental approaches showed significant correlations (Gogina et al., 2017; Morys, Powilleit & Forster, 2017; Wrede et al., 2017), which supports the use of BP_c_.

There are also limitations on the use of BP_c_ as an indicator in combination with projected changes of environmental variable and expected changes in ecosystem functioning. We did not account for changes in sediment structure or current regime, which were kept constant in the distribution modelling (Weinert et al., 2021). It is well known that some environmental parameters that can affect the spatial distribution of species in the North Sea often change simultaneously, such as temperature and food availability, which was not taken into account in our study either. Some studies though have already demonstrated temperature-related changes in the community structure and bioturbation potential of benthic organisms (Reiss, Meybohm & Kröncke, 2006; Clare et al., 2017; Meyer, Nehmer & Kröncke, 2019), which we utilize in our modelling approach. In addition to the geographical distribution of the species, temperature also regulates the metabolic rates (Brown et al., 2004). On the one hand, a combination of high temperatures and low food availability may lead to a reduced bioturbation due to the need to save energy and the concerning BP_c_ would be an overestimation (Maire et al., 2007, 2008). On the other hand, temporarily high temperatures can lead to higher bioturbation intensity due to increased activity, while a lower food availability may lead to increased bioturbation activity to ensure the uptake of enough organic carbon. Both processes would lead to an underestimated BP_c_. Indeed bioturbation is more strongly linked to the metabolic rate than abundance due to the mechanistic relationship with respiration, feeding and moving (Cozzoli et al., 2021). Although metabolic rates were not considered in our study, the modelled BP is a product of abundance and biomass, with the latter more closely linked to the metabolism of organisms. However, the approach accounts for the direction and intensity of distributional changes of bioturbators as an important driver of changes in bioturbation (or biotransport in general) and the consecutive ecosystem functions (Cozzoli et al., 2021).

## Conclusion

Our results indicate that the selected bioturbators of the southern North Sea might balance negative regional effects of increasing temperature on bioturbation potential through a northward territorial expansion. This leads to a decrease of BP_p_ for several species in former core regions, but a wider spatial distribution with low or intermediate BP_p_. One of the most important bioturbators in the North Sea, the sea urchin *Echinocardium cordatum*, showed relatively constant BP_p_ values until 2099, although a reduction of suitable habitat was projected due to temperature and salinity changes (Weinert et al., 2016), while the BP_p_ of other key species such as the brittle star *Amphiura filiformis* or *Nucula nitidosa* declined especially in their core distribution in the southern North Sea. Thus, the relative contribution of the species to the BP was projected to change in the southern North Sea. It remains speculative to what extent functional redundancy among species in the community can actually buffer against these changes in species contribution to bioturbation and consequently ecosystem functioning. It seems like species are not easily substitutable due to subtle differences in their activity and behaviour (Hale et al., 2014). Indeed, while *E. cordatum* was found to be the most important bioturbator in the North Sea, not only based on high BP_p_ values but also due to their actually burrowing activities, *A. filiformis* with similar high BP_p_ was found to be a less active bioturbator (Wrede et al., 2017). Thus, species that were not accounted for in our study might play a larger role in shaping ecosystem processes under warming conditions, which might also be amplified by temperature effects on activity and metabolism of organisms (Cozzoli et al., 2021).

Moreover, BP_p_ is strongly dependent on macrofaunal biomass, which is in turn affected by the de-eutrophication processes and the decline in primary production observed in the southern North Sea in the last decades (Boersma et al., 2015; Meyer et al., 2018; van Beusekom et al., 2019; Desmit et al., 2020; Xu, Lemmen & Wirtz, 2020). If this trend continues, it might lead to an even stronger decline in BP in the southern North Sea over the next decade than projected for temperature and salinity changes alone as in our study.

## Supplemental Information

10.7717/peerj.14105/supp-1 Supplemental Information 1 Mean bottom temperatures [°C] (a, b, d, e, g, h) and salinity (c, f, i) in the North Sea for February and June 2001, 2050 and 2099, and as well the differences between the years (j-o). Diff = difference, black line: 50 m depth contour. The projected mean bottom temperature and salinity for 2050 and 2099 are based on IPCC A1B scenario (modified after Weinert et al., 2016).

10.7717/peerj.14105/supp-2 Supplemental Information 2 General work-flow of the two step approach in the study. First, Species Distribution Models (biomod2) were applied, to model and project the species distribution (po = species probability of occurrence, p = species presence, a = species absence.) Second, Random Forest was applied to model and project the species bioturbation potential (BP).

10.7717/peerj.14105/supp-3 Supplemental Information 3 R script to model and project the species data.

## Additional Information and Declarations

### Data Availability

The following information was supplied regarding data availability: The code for analysis is available in the Supplemental Files. We used proprietary data for the analysis and can be requested from the following sources: The species data is available at MAFCONS: http://www.vliz.be/projects/mafcons/ Environmental data: - Temperature and salinity: Moritz Mathis, Helmholtz-Zentrum Hereon, Institute of Coastal Systems, Max-Planck-Straße 1, 21502 Geesthacht, Germany (moritz.mathis@hereon.de). AND Thomas Pohlmann, Institute of Oceanography, University of Hamburg, Bundesstr. 53, 20146 Hamburg, Germany (thomas.pohlmann@uni-hamburg.de). - Mud content and median grain size: Please contact a member of the ICES North Sea Benthos Project 2000 (http://www.vliz.be/vmdcdata/nsbp) or a colleague from the EU Project MAFCONS (http://www.vliz.be/projects/mafcons). - Peak wave stress: Judith Wolf, National Oceanography Centre, Joseph Proudman Building, 6 Brownlow Street, Liverpool L3 5DA, UK (jaw@noc.ac.uk). - Depth: The data is public at The General Bathymetric Chart of the Oceans (GEBCO): https://www.gebco.net.
