# Supplementary material for: Benthic ecosystem functioning under climate change: modelling the bioturbation potential for benthic key species in the southern North Sea
Source: PeerJ. 2022 Oct 26;10:e14105. doi: 10.7717/peerj.14105 (PMC9617549; doi:10.7717/peerj.14105)
Supplement: Supplemental Information 1 — Diff = difference, black line: 50 m depth contour. The projected mean bottom temperature and salinity for 2050 and 2099 are based on IPCC A1B scenario (modified after Weinert et al., 2016). [file peerj-10-14105-s001.pdf]

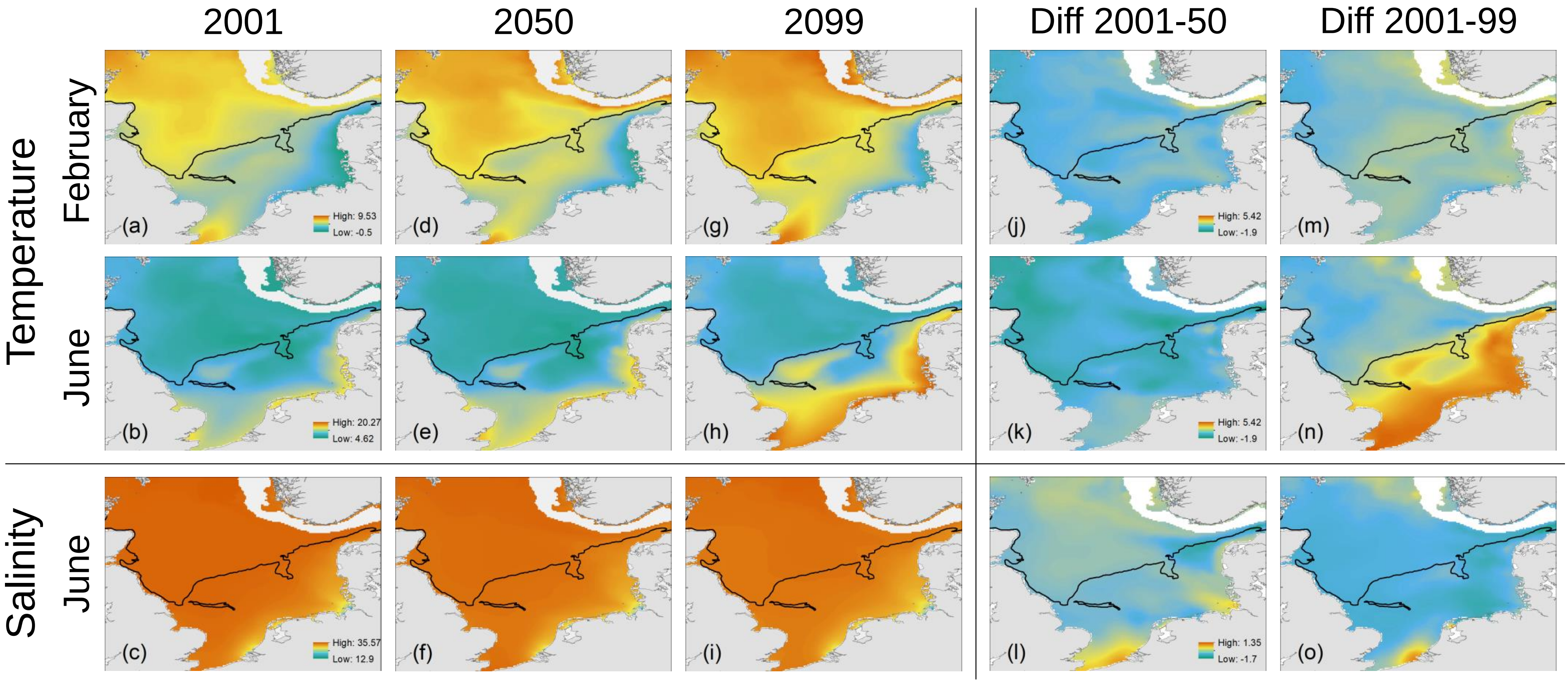

S1 Mean bottom temperatures [°C] (a, b, d, e, g, h) and salinity (c, f, i) in the North Sea for February and June 2001, 2050 and 2099, and as well the differences between the years (j-o). Diff = difference, black line: 50 m depth contour. The projected mean bottom temperature and salinity for 2050 and 2099 are based on IPCC A1B scenario (modified after *Weinert et al. 2016*).
