# Supplementary material for: Benthic ecosystem functioning under climate change: modelling the bioturbation potential for benthic key species in the southern North Sea
Source: PeerJ. 2022 Oct 26;10:e14105. doi: 10.7717/peerj.14105 (PMC9617549; doi:10.7717/peerj.14105)
Supplement: Supplemental Information 2 — First, Species Distribution Models (biomod2) were applied, to model and project the species distribution (po = species probability of occurrence, p = species presence, a = species absence.) Second, Random Forest was applied to model and project the species bioturbation potential (BP). [file peerj-10-14105-s002.pdf]

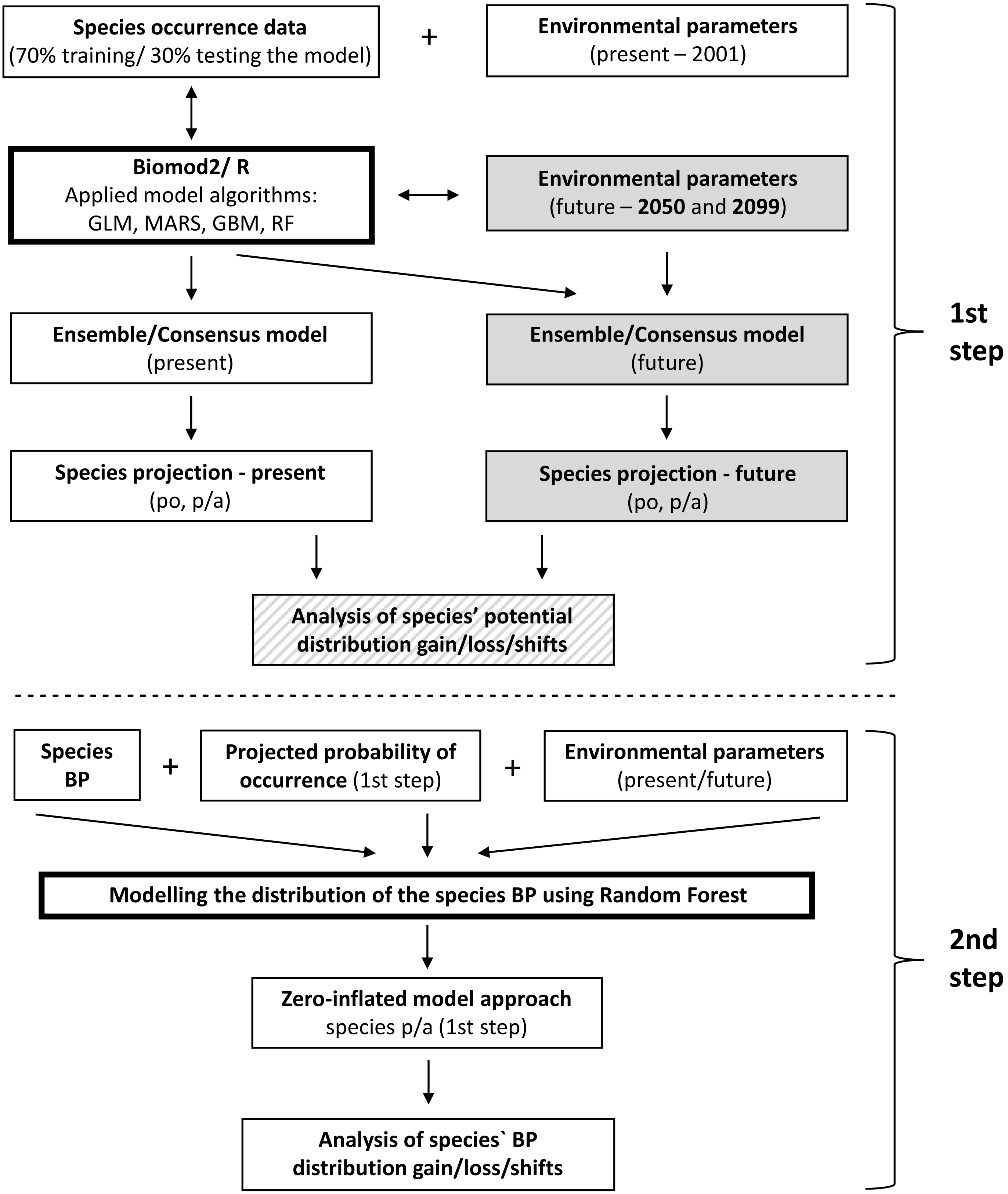

S2 Shows the general work-flow of the two step approach. First, Species Distribution Models (biomod2) were applied, to model and project the species distribution (po = species probability of occurrence, p = species presence, a = species absence.) Second, Random Forest was applied to model and project the species bioturbation potential (BP).
